# Beneficial Effects of Concentrated Growth Factors and Resveratrol on Human Osteoblasts* In Vitro* Treated with Bisphosphonates

**DOI:** 10.1155/2018/4597321

**Published:** 2018-05-16

**Authors:** Elisa Borsani, Veronica Bonazza, Barbara Buffoli, Pier Francesco Nocini, Massimo Albanese, Francesca Zotti, Francesco Inchingolo, Rita Rezzani, Luigi F. Rodella

**Affiliations:** ^1^Department of Clinical and Experimental Sciences, Division of Anatomy and Physiopathology, University of Brescia, Brescia, Italy; ^2^Interdipartimental University Center of Research “Adaption and Regeneration of Tissues and Organs (ARTO)”, University of Brescia, Brescia, Italy; ^3^Department of Surgery, Dentistry, Pediatric and Gynecology, Division of Oral and Maxillofacial Surgery, University of Verona, Verona, Italy; ^4^Department of Interdisciplinary Medicine, University of Bari “Aldo Moro”, Bari, Italy

## Abstract

Bisphosphonates are primary pharmacological agents against osteoclast-mediated bone loss and widely used in the clinical practice for prevention and treatment of a variety of skeletal conditions, such as low bone density and osteogenesis imperfecta, and pathologies, such as osteoporosis, malignancies metastatic to bone, Paget disease of bone, multiple myeloma, and hypercalcemia of malignancy. However, long-term bisphosphonate treatment is associated with pathologic conditions including osteonecrosis of the jaw, named BRONJ, which impaired bone regeneration process. Clinical management of BRONJ is controversy and one recent approach is the use of platelet concentrates, such as Concentrated Growth Factors, alone or together with biomaterials or antioxidants molecules, such as resveratrol. The aim of the present study was to investigate the* in vitro* effects of Concentrated Growth Factors and/or resveratrol on the proliferation and differentiation of human osteoblasts, treated or not with bisphosphonates. Human osteoblasts were stimulated for 3 days in complete medium and for 21 days in mineralization medium. At the end of the experimental period, the* in vitro* effect on osteoblast proliferation and differentiation was evaluated using different techniques such as MTT, ELISA for the quantification/detection of osteoprotegerin and bone morphogenetic protein-2, immunohistochemistry for sirtuin 1 and collagen type I, and the Alizarin Red S staining for the rate of mineralization. Results obtained showed that Concentrated Growth Factors and/or resveratrol significantly increased osteoblast proliferation and differentiation and that the cotreatment with Concentrated Growth Factors and resveratrol had a protective role on osteoblasts treated with bisphosphonates. In conclusion, these data suggest that this approach could be promised in the clinical management of BRONJ.

## 1. Introduction

Bisphosphonates are primary pharmacological agents against osteoclast-mediated bone loss promoting the apoptosis of osteoclasts actively engaged in the degradation of mineral on the bone surface. Bisphosphonates, such as zoledronic acid or zoledronate, alendronate, and pamidronate, are important inhibitors of bone resorption and are widely used for managing a variety of skeletal conditions characterized by increased osteoclast-mediated bone resorption, including multiple forms of osteoporosis (e.g., juvenile, postmenopausal, or senile), osteogenesis imperfecta, Paget disease of bone, hypercalcemia, and malignancy metastatic to bone. However, long-term bisphosphonate treatment is associated with a rare but serious adverse event: osteonecrosis of the maxillary and mandibular bones [1–3]. This condition is called bisphosphonate-related osteonecrosis of the jaw or BRONJ. It can be described as a condition in which an area of jawbone is not covered by the gums. The condition must last for more than eight weeks in a person taking any bisphosphonate. When the bone is left uncovered, it does not receive blood and begins to die. Symptoms of BRONJ include exposed bone, localized pain, swelling of the gum tissues and inflammation, and loosening of previously stable teeth. The mechanism underlying BRONJ has proved difficult to unravel and the three broad categories of intervention include conservative approaches (e.g., mouth rinse, antibiotics), surgical interventions, and adjuvant nonsurgical strategies (e.g., hyperbaric oxygen therapy, platelet concentrates, and laser application), which can be used in combination [4]. All these approaches aim at bone regeneration, which can be required in large quantity. Bone regeneration is a complex, well-orchestrated physiological process of bone formation, which involves different factors, a number of cell types, and intracellular and extracellular molecular-signaling pathways, with a definable temporal and spatial sequence, in order to optimize skeletal repair and restore skeletal function.

Among the different strategies used to augment the impaired or “insufficient” bone regeneration process, one current approach is the local application of growth factors through the use of platelet concentrates, such as Concentrated Growth Factors (CGF) [5, 6]. They are autologous preparations obtained from the patient's own blood that is then subjected to a specific and standardized protocol of centrifugation [7, 8]. CGF represents the most recent concentrate, developed by Sacco in 2006. Several data suggest that it possesses a good regenerative capacity and versatility, having a positive effect for sinus and alveolar ridge augmentation [9], preimplant augmentation procedures [10], promotion of* in vitro* proliferation, osteogenic maturation and mineralization of mesenchymal stem cells, and healing of critical-size bone defects* in vivo* [11], and promotion of* in vitro* periodontal ligament stem cells proliferation [12]. The efficacy of this treatment lies in the local delivery of a wide range of growth factors and proteins, mimicking and supporting physiologic wound healing, reparative tissue process, and local, infiltration therapy. CGF can be used alone or mixed with biomaterials such as collagen, hydroxyapatite (HA), *β*-tricalcium phosphate (*β*-TCP), calcium-phosphate cements, and also with antioxidant molecules. Among these molecules, resveratrol is generally known to be beneficial for human health, due to its anti-inflammatory, antioxidant effects, and bone protective properties [13], that are mediated via the activation of sirtuin 1 (SIRT-1) [14]. It is a polyphenolic compound that occurs naturally in mulberries, peanuts, grape and berry skins, and red wine [15, 16]. Several biological effects of resveratrol have been reported, including platelet aggregation attenuation [17], cardiovascular protection [18, 19], anticancer activity [20, 21], and neuroprotective effect [22]. Other studies show that resveratrol stimulates* in vitro* osteoblast cells proliferation and differentiation [15, 23, 24] and prevented osteoporosis in ovariectomized rats [25, 26] and steroid-induced osteonecrosis in a rabbit model [27].

Based on these considerations, the growth factors delivered by CGF and the antioxidant effect of resveratrol could have a synergic role in bone deposition and protection against the side effects of bisphosphonates. So, the aim of the present study was to investigate the* in vitro* effect of treatment with CGF and/or resveratrol on the proliferation (3 days) and differentiation (21 days) of human osteoblast cells (HOB), treated or not with bisphosphonates monitoring indicative molecular recognised markers, such as osteoprotegerin (OPG) [28], bone morphogenetic protein-2 (BMP-2) [29], collagen type I (Col I) [30], sirtuin 1 (SIRT-1) [31], and Alizarin Red S staining [32] for calcium deposits.

## 2. Materials and Methods

### 2.1. Cell Culture

Human osteoblasts (HOB, PromoCell, Heidelberg, Germany) were grown in Osteoblast Growth Medium (OGM; PromoCell, Heidelberg, Germany) constituted by Osteoblast Basal Medium (OBM; PromoCell, Heidelberg, Germany) supplemented with gentamicin/amphotericin B (antibiotic/antifungal) and Supplement Mix (OGM Supplement Mix; PromoCell, Heidelberg, Germany) containing growth factors (not specified by the manufacturer) at 37°C, 5% CO_2_, in a humidified atmosphere until they reached about 80% confluence, changing the medium every 2 days. Experiments were performed using cells between passages 3-4. At confluence, HOB were passaged and seeded in 6-well culture plates (Sarstedt, Nuembrecht, Germany). For short time experiments (3 days), the cells were starved in OBM, free of serum and growth factors, for 24 hours; at the end of the starvation period, the medium was removed and the different cell treatments were performed (Tables 1 and 2). For long time experiments (21 days), the OGM was replaced with the Osteoblast Mineralization Medium (OMM; PromoCell, Heidelberg, Germany) and the cells were subjected to different treatments (Table 3). The* in vitro* experiments were performed in triplicate to ensure the reproducibility of results.

### 2.2. Resveratrol and Bisphosphonates Preparation

Resveratrol with purity greater than 98% was purchased from Sigma-Aldrich (Milan, Italy). A 1 mM stock solution of resveratrol was prepared in absolute ethanol and further diluted in cell culture medium to prepare the final working concentration of 10 *μ*M, determined by a preliminary evaluation of different concentration (1, 5, 10, 25, and 50 *μ*M) on cell viability using MTT assay (Table 1) [33]. The maximum final content of ethanol in cultures was less than 0,1%. As regards bisphosphonates, based on literature data, we chose alendronate (AL) and zoledronate or zoledronic acid (ZOL). Both AL and ZOL were purchased from Sigma-Aldrich (Milan, Italy), in a sterile powder form. They were diluted in cell culture medium to obtain the final working concentration of 5 *μ*M [34]. All the solutions were filtered under a flow laminar cabinet before the use.

### 2.3. CGF Preparation

CGF was obtained by a venous blood sampling from three male adult volunteers, who gave their previous written consent, with no systemic disorders, smoking, infections, nonsteroidal anti-inflammatory drug use, and a haemoglobin level < 13,5 g/dl. Blood (9 ml) was drawn in each sterile Vacuette tube (Greiner Bio-One, GmbH, Kremsmünster, Austria) silicon coated as serum clot activator. These tubes were then immediately centrifuged using a special machine (Medifuge MF200, Silfradent S.r.l., Forlì, Italy). At the end of the centrifugation process, three blood fractions were identified: (1) the upper layer, representing the liquid phase of plasma named platelet poor plasma (PPP); (2) the lower layer, at the bottom of the tube, consisting in free red blood cells (RBC); (3) the middle layer, representing the solid CGF, consisting in three fractions: the upper white part (WP), the downer red part (RP), and the middle “buffy coat” (BC), interface between white and red part [7, 8]. After centrifugation, each CGF was removed from the tube, using sterile tweezers, under a laminar flow cabinet. The solid CGF was obtained by cutting and discarding the lower fraction of the red part of CGF. Subsequently, each CGF was processed in relation to the experimental protocols, as described in detail in the next paragraphs. The* in vitro* experiments were performed in triplicate to ensure the reproducibility of results.

### 2.4. MTT Colorimetric Assay

At the end of the different treatment performed for 3 days, MTT (Thiazolyl Blue Tetrazolium Blue, Sigma-Aldrich, Milan, Italy, 5 mg/ml) was added to each well for 4 hours at 37°C. The culture medium was aspirated and the same quantity of DMSO was added to each well. The plate was put on the shaker for 15 minutes and after the supernatant was transferred to a 96-microplate well. Colorimetric changes were quantified in a microplate reader (Tecno-Lab S.r.l., Brescia, Italy) at a wavelength of 540 nm. The MTT assay was performed in triplicate and data obtained were expressed as the percentage optical density relative to the negative control of 100%.

### 2.5. Enzyme Linked Immunosorbent Assay (ELISA)

To assess the possible effect of resveratrol and/or CGF on osteoblasts treated or not with bisphosphonates, a human osteoprotegerin (OPG) ELISA kit (LifeSpan Biosciences, Inc., Seattle, USA) and a human bone morphogenetic protein-2 (BMP-2) ELISA kit (R&D Systems Inc., Minneapolis, Minnesota, USA) were used to detect, respectively, the levels of OPG and the levels of BMP-2, in the cell supernatant. Briefly, cells were seeded in 6 well plates and subjected to different treatments, at different times, as reported in Tables 2 and 3. At the end of the different treatments, the conditioned medium was collected, centrifuged for 10 min at 1000 ×g, and stored at −80°C until the OPG and BMP-2 concentration was measured using the specific ELISA kit, according to the manufacturer's instructions.

### 2.6. Immunohistochemistry for Sirtuin 1 (SIRT-1) and Collagen Type I (Col I)

After the diverse treatments, their effect on the sirtuin 1 (SIRT-1) expression and collagen type I (Col I) production was investigated by immunohistochemistry after 3 days of culture. The UltraVision™ Quanto Detection System Horseradish Peroxidase (HRP; ThermoScientific™, Thermo Fischer Scientific, CA, USA) followed by development with the chromogen substrate 3,3′-Diaminobenzidine (DAB, Amresco, Prodotti Gianni, Milan, Italy) was used to perform immunohistochemistry. Before adding the mouse monoclonal antibodies SIRT-1 and Col I (Santa Cruz Biotechnology, Texas, USA), the cells were permeabilized with Triton 0,1% for 10 minutes and then incubated with blocking solution for 5 minutes before adding the specific primary antibody at appropriate dilution, for 1 hour, at room temperature. Subsequently, the primary antibody enhancer was added for 10 minutes and lastly the HRP Polimer Quanto for 10 minutes. The chromogen substrate DAB was added to the cells, monitoring staining closely. After dehydration and mounting with DPX, cells were detected using light microscopy. Digitally fixed images were produced and analyzed using a dedicated image software (Image Pro-Premier, Immagini & Computer, Milan, Italy) and the Integrated Optical Density (IOD) was measured and normalized to cell number [30].

### 2.7. Alizarin Red S Staining

The degree of calcium deposition on human osteoblasts (HOB) cultured in mineralization medium (OMM) for 21 days was determined using Alizarin Red S staining. At the end of the treatment period, the culture medium was removed and HOB were washed twice with phosphate buffered saline (PBS) and fixed with 4% paraformaldehyde for 15 min. The fixed cells were then stained with Alizarin Red S staining solution (Sigma-Aldrich, Milan, Italy), for 45 min, at room temperature in the dark. Then the Alizarin Red S staining solution was aspirated and cells were washed four times with distilled water. The stained cells were dehydrated and mounted with DPX (Sigma-Aldrich, Milan, Italy), for light microscopy detection in order to quantify the amount of calcium deposits. Undifferentiated HOB (without extracellular calcium deposits) were slightly reddish, whereas mineralized osteoblasts (with extracellular calcium deposits) were bright orange-red. Digitally fixed images of cells were analyzed using an image analyzer (Image Pro-Premier, Immagini & Computer, Milan, Italy) and the Integrated Optical Density (IOD) was measured and normalized to cell number [30].

### 2.8. Statistical Analysis

The data collected were analyzed by one-way ANOVA followed by Bonferroni test. The level of significance was set at 5% (*p* < 0.05).

## 3. Results

### 3.1. MTT Colorimetric Assay Results

**Figure 1:**
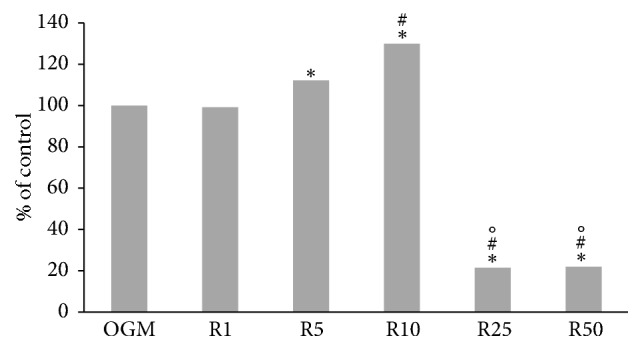
MTT assay after 3 days of treatment with different concentration of resveratrol for the determination of working concentration: 1 *μ*M (R1), 5 *μ*M (R5), 10 *μ*M (R10), 25 *μ*M (R25), and 50 *μ*M (R50) in complete medium (OGM), which represent the control. Data represent the percentage of control, ^*∗*^*p* < 0.05 versus OGM, ^#^*p* < 0.05 versus R5, and °*p* < 0.05 versus R10.

MTT after 3 days of treatment with different concentration of resveratrol showed that the best concentration was 10 *μ*M, which was the highest concentration without cytotoxic effects and so used for the subsequent experiments (Figure 1).

**Figure 2:**
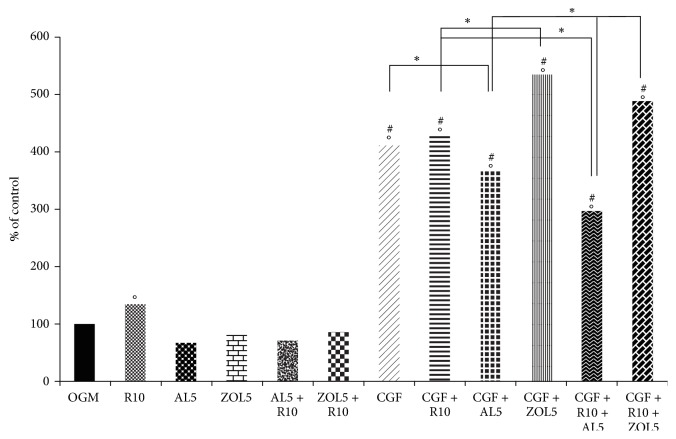
MTT assay after 3 days of treatment: resveratrol 10 *μ*M (R10), alendronate 5 *μ*M (AL5), zoledronate 5 *μ*M (ZOL5), alendronate 5 *μ*M and resveratrol 10 *μ*M (AL5 + R10), and zoledronate 5 *μ*M and resveratrol 10 *μ*M (ZOL5 + R10) in complete medium (OGM), which represent the control; Concentrated Growth Factors (CGF): CGF and resveratrol 10 *μ*M (CGF + R10), CGF and alendronate 5 *μ*M (CGF + AL5), CGF and zoledronate 5 *μ*M (CGF + ZOL5), CGF and alendronate 5 *μ*M and resveratrol 10 *μ*M (CGF + R10 + AL5), and CGF and zoledronate 5 *μ*M and resveratrol 10 *μ*M (CGF + R10 + ZOL5). Data represent the percentage of control, ^*∗*^*p* < 0,05 and in particular °*p* < 0,05 versus OGM; ^#^*p* < 0,05 versus R10, AL5, ZOL5, AL5 + R10, ZOL5 + R10.

MTT after 3 days of treatment with resveratrol 10 *μ*M (R10), CGF and bisphosphonates alone and used in combination, showed different results (Figure 2). Treatment with R10 significantly affected osteoblast proliferation (133,1%) with respect to complete medium (OGM) alone (100%), which represents the control. On the contrary, treatment with alendronate 5 *μ*M (AL5: 67,3%), zoledronate 5 *μ*M (ZOL5: 80,4%), AL5 + R10 (69,5%), and ZOL5 + R10 (85%) did not significantly increase osteoblasts proliferation with respect to OGM alone or supplemented with R10. With the use of CGF, alone and combined with R10 and/or bisphosphonates, the results significantly changed. In fact, treatments with CGF (410,8%), CGF + R10 (428,4%), and CGF + AL5 (367,4%) significantly increased cell proliferation and those were statistically different compared with OGM, R10, AL5, ZOL5, AL5 + R10, and ZOL5 + R10. Costimulation with CGF + ZOL5 showed the highest percentage of cell vitality (535,3%) and it was statistically different with respect to all previous treatments with the exclusion of CGF, CGF + R10, and CGF + AL5. In CGF + R10 + AL5, the percentage of cell vitality was slower (296,2%) with respect to the other treatments with CGF but it was statistically different with respect to all treatments. On the contrary, using CGF and R10 in the presence of ZOL5 (CGF + R10 + ZOL5), cell proliferation significantly increased (487,4%), being statistically different with respect to all treatments, except CGF, CGF + R10, and CGF + ZOL5.

### 3.2. Enzyme Linked Immunosorbent Assay (ELISA) for Osteoprotegerin (OPG) and Bone Morphogenetic Protein-2 (BMP-2)

**Figure 3:**
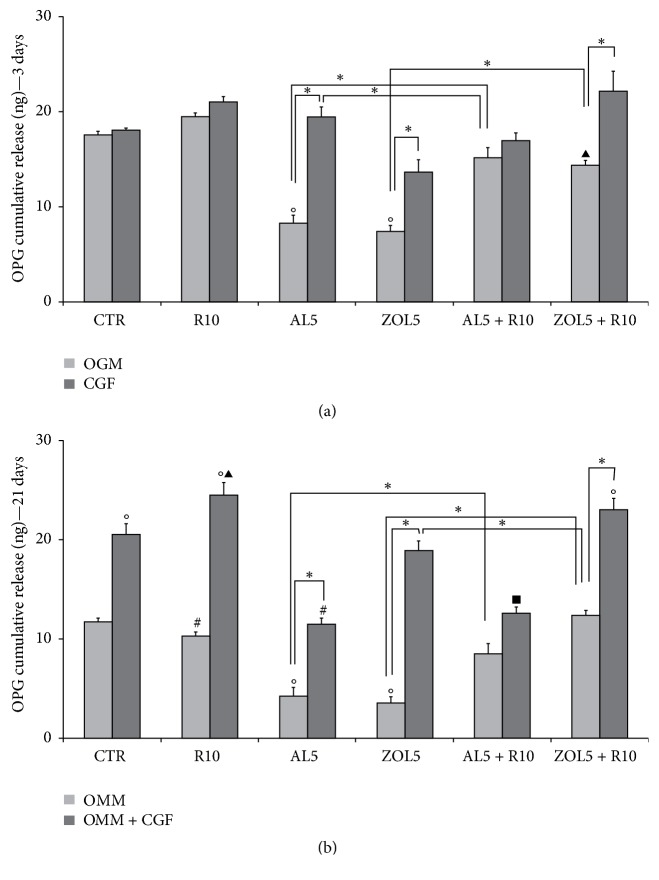
Cumulative osteoprotegerin (OPG, ng) release after 3 days (a) and 21 days (b) of culture. (a) The cells were cultured in complete medium (OGM) or Concentrated Growth Factor (CGF), which represents the controls, and in presence of resveratrol 10 *μ*M (R10), alendronate 5 *μ*M (AL5), zoledronate 5 *μ*M (ZOL5), alendronate 5 *μ*M and resveratrol 10 *μ*M (AL5 + R10), and zoledronate 5 *μ*M and resveratrol 10 *μ*M (ZOL5 + R10). The values are reported as total amount of OPG released ± SE, ^*∗*^*p* < 0,05 and in particular °*p* < 0,05 versus OGM; ^▴^*p* < 0,05 versus OGM + R10. (b) The cells were cultured in mineralization medium (OMM) also supplemented with Concentrated Growth Factor (OMM + CGF), which represents the controls, and in presence of resveratrol 10 *μ*M (R10), alendronate 5 *μ*M (AL5), zoledronate 5 *μ*M (ZOL5), alendronate 5 *μ*M and resveratrol 10 *μ*M (AL5 + R10), and zoledronate 5 *μ*M and resveratrol 10 *μ*M (ZOL5 + R10). The values are reported as total amount of OPG released ± SE, ^*∗*^*p* < 0,05 and in particular °*p* < 0,05 versus OMM; ^#^*p* < 0,05 versus OMM + CGF; ^▴^*p* < 0,05 versus OMM + R10; ^*▪*^*p* < 0,05 versus OMM + CGF + R10.

**Figure 4:**
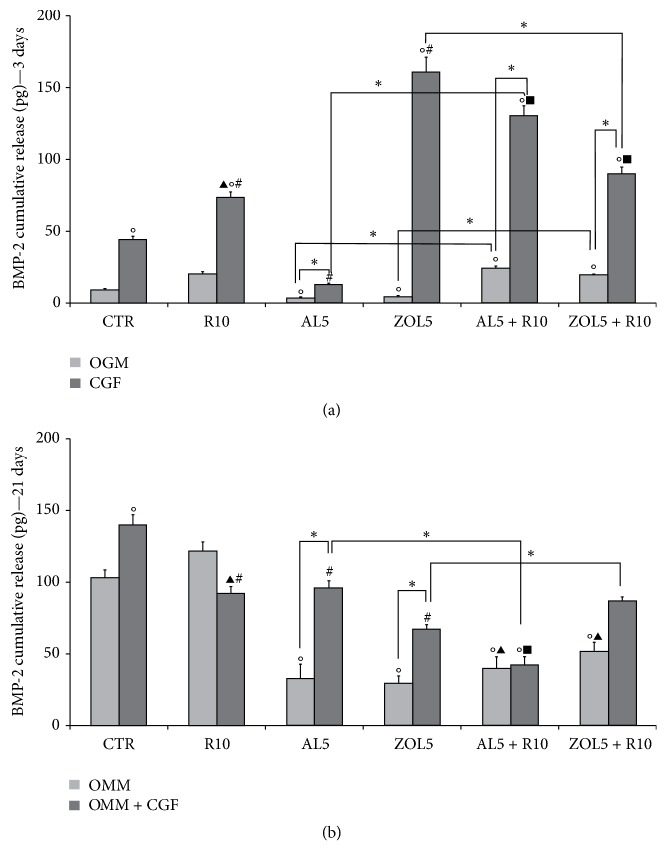
Cumulative bone morphogenetic protein-2 (BMP-2, pg) release after 3 days (a) and 21 days (b) of culture. (a) The cells were cultured in complete medium (OGM) or Concentrated Growth Factor (CGF), which represents the controls, and in presence of resveratrol 10 *μ*M (R10), alendronate 5 *μ*M (AL5), zoledronate 5 *μ*M (ZOL5), alendronate 5 *μ*M and resveratrol 10 *μ*M (AL5 + R10), and zoledronate 5 *μ*M and resveratrol 10 *μ*M (ZOL5 + R10). The values are reported as total amount of BMP-2 released ± SE, ^*∗*^*p* < 0,05 and in particular °*p* < 0,05 versus OGM; ^#^*p* < 0,05 versus CGF; ^▴^*p* < 0,05 versus OGM + R10; ^*▪*^*p* < 0,05 versus CGF + R10. (b) The cells were cultured in mineralization medium (OMM) also supplemented with Concentrated Growth Factor (OMM + CGF), which represents the controls, and in presence of resveratrol 10 *μ*M (R10), alendronate 5 *μ*M (AL5), zoledronate 5 *μ*M (ZOL5), alendronate 5 *μ*M and resveratrol 10 *μ*M (AL5 + R10), and zoledronate 5 *μ*M and resveratrol 10 *μ*M (ZOL5 + R10). The values are reported as total amount of BMP-2 released ± SE, ^*∗*^*p* < 0,05 and in particular °*p* < 0,05 versus OMM; ^#^*p* < 0,05 versus OMM + CGF; ^▴^*p* < 0,05 versus OMM + R10; ^*▪*^*p* < 0,05 versus OMM + CGF + R10.

ELISA for osteoprotegerin (OPG) (Figures 3(a) and 3(b)) and bone morphogenetic protein-2 (BMP-2) (Figures 4(a) and 4(b)) after 3 and 21 days of culture showed different results. Both OPG and BMP-2 showed a different trend after 3 days in OGM (complete medium) and 21 days in OMM (mineralization medium).

As regards OPG after 3 days (Figure 3(a)), its levels increased in cells treated with resveratrol 10 *μ*M (R10: 19,50 ng ± 0,70), CGF + R10 (21,03 ng ± 0,4), and CGF + alendronate 5 *μ*M (AL5) (19,5 ng ± 1,80), with respect to untreated cells, cultured in complete medium alone (OGM: 17,59 ng ± 0,80). Treatment with CGF + zoledronate 5 *μ*M (ZOL5) led to a significant decrease (13,70 ng ± 2,30) with respect to R10, CGF + R10, and CGF + AL5. In CGF + R10 + AL5, OPG levels slightly increased (16,90 ng ± 1,50) compared with CGF + ZOL5 but they were lower with respect to the other experimental conditions. On the contrary, the highest OPG concentration was observed in cells treated with CGF + R10 + ZOL5 (22,3 ng ± 3,70) and it was statistically significant with respect to CGF + ZOL5.

After 21 days in mineralization medium (OMM), OPG levels showed a different trend (Figure 3(b)), reaching a significant increase in cells treated with CGF (20,50 ng ± 1,80), CGF + R10 (24,50 ng ± 1,3), R10 + ZOL5 (18,90 ng ± 1,90), and CGF + R10 + ZOL5 (23,03 ng ± 2,00). All these treatments were statistically different compared with control, OMM (11,70 ng ± 1,1) and OMM + R10 (10,30 ng ± 0,90). Moreover, treatments with OMM + R10 + ZOL5 and OMM + CGF + R10 + ZOL5 were statistically different also compared with OMM + CGF + R10 and OMM + R10 + ZOL5. Treatments with OMM + R10 + AL5 (11,50 ng ± 1,05) and OMM + CGF + R10 + ZOL5 (12,60 ng ± 1,15) led to a significant decrease in OPG levels, compared with OMM + CGF, OMM + CGF + R10, and in the case of the second treatment (OMM + CGF + R10 + ZOL5), also with OMM + R10 + ZOL5.

As regards BMP-2, after 3 days of culture (Figure 4(a)) its levels progressively increased in cells treated with CGF (44,00 pg ± 4,00) and with CGF + R10 (73,30 pg ± 6,65). In fact, both treatments were statistically different with respect to OGM (9,13 pg ± 0,85) and costimulation with CGF + R10 resulted statistically different also compared with R10 (20,13 pg ± 1,85). Cotreatment with CGF + AL5 led to a significant decrease in BMP-2 levels (12,7 pg ± 1,21) with respect to CGF + R10. On the contrary, cotreatment with CGF + ZOL5 led to the highest increase in BMP-2 levels (203,50 pg ± 18,50), being statistically different with respect to OGM, R10, CGF, CGF + R10, and CGF + AL5. Also, costimulation with CGF + R10 + AL5 led to a significant increase in BMP-2 levels being statistically different compared with all treatments. Similarly, cotreatment with CGF + R10 + ZOL5 significantly increased BMP-2 levels, being statistically different with respect to all treatments, except that with CGF + R10.

After 21 days of culture in mineralization medium (OMM), BMP-2 showed a different trend (Figure 4(b)) with respect to that after 3 days in OGM. BMP-2 levels progressively increased reaching a significant augment after treatment with OMM + CGF (139,20 pg ± 12,70) that was statistically different with respect to OMM (102,70 pg ± 9,30) and OMM + CGF (121 pg ± 11). Cotreatment with OMM + CGF + R10 led to a significant decrease (91,60 pg ± 8,35) in BMP-2 levels, with respect to OMM + R10 and OMM + CGF. Similarly, also costimulation with OMM + R10 + AL5 significantly reduced BMP-2 levels (95,40 pg ± 8,70) being statistically different compared with OMM + CGF. Also, treatment with OMM + R10 + ZOL5 significantly decreased BMP-2 levels (56,80 pg ± 5,20) and this reduction was statistically different with respect to all treatments. On the contrary, after costimulation with OMM + CGF + R10 + AL5, BMP-2 levels increased (111,90 pg ± 10,00), being statistically significant compared with OMM + R10 + ZOL5. Using CGF + R10 + ZOL5, BMP-2 levels slightly decreased with respect to OMM + CGF + R10 + AL5 (86,36 pg ± 4,70), being statistically different with respect to OMM + R10, OMM + CGF and OMM + R10 + ZOL5.

### 3.3. Immunohistochemistry for Sirtuin 1 (SIRT-1) and Collagen Type I (Col I)

**Figure 5:**
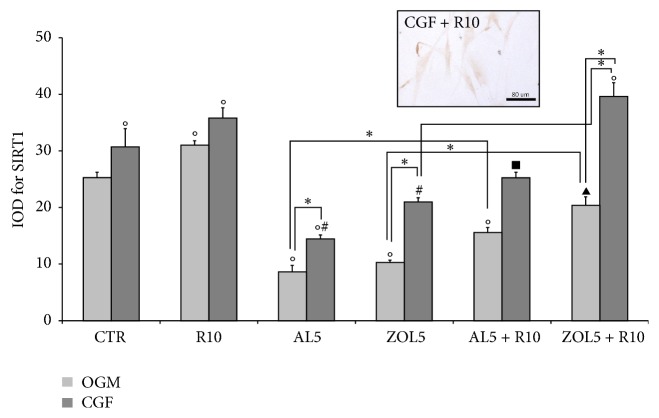
Quantitative evaluation of sirtuin 1 (SIRT-1) immunopositivity in osteoblasts (HOB) after 3 days of culture as IOD (Integrated Optical Density). A micrograph as an example has been reported. The cells were cultured in complete medium (OGM) or Concentrated Growth Factor (CGF), which represents the controls, and in presence of resveratrol 10 *μ*M (R10), alendronate 5 *μ*M (AL5), zoledronate 5 *μ*M (ZOL5), alendronate 5 *μ*M and resveratrol 10 *μ*M (AL5 + R10), and zoledronate 5 *μ*M and resveratrol 10 *μ*M (ZOL5 + R10). Data represent means ± SE, ^*∗*^*p* < 0,05 and in particular °*p* < 0,05 versus OGM; ^#^*p* < 0,05 versus CGF; ^▴^*p* < 0,05 versus OGM + R10; ^*▪*^*p* < 0,05 versus CGF + R10.

**Figure 6:**
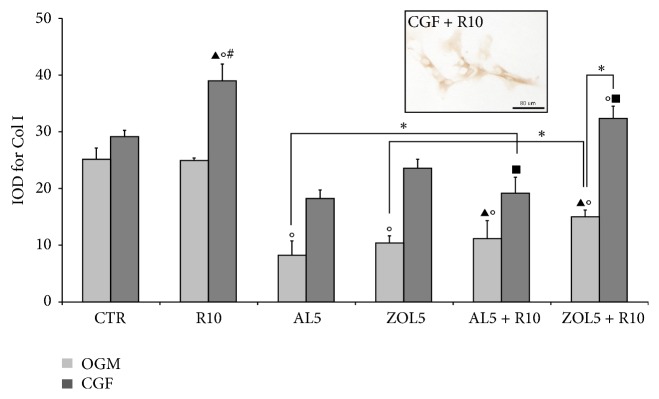
Quantitative evaluation of collagen type I (Col I) immunopositivity in osteoblasts (HOB) after 3 days of culture as IOD (Integrated Optical Density). A micrograph as an example has been reported. The cells were cultured in complete medium (OGM) or Concentrated Growth Factor (CGF), which represents the controls, and in presence of resveratrol 10 *μ*M (R10), alendronate 5 *μ*M (AL5), zoledronate 5 *μ*M (ZOL5), alendronate 5 *μ*M and resveratrol 10 *μ*M (AL5 + R10), and zoledronate 5 *μ*M and resveratrol 10 *μ*M (ZOL5 + R10). Data represent means ± SE, ^*∗*^*p* < 0,05 and in particular °*p* < 0,05 versus OGM; ^#^*p* < 0,05 versus CGF; ^▴^*p* < 0,05 versus OGM + R10; ^*▪*^*p* < 0,05 versus CGF + R10.

Immunohistochemistry for sirtuin 1 (SIRT-1) (Figure 5) and collagen type I (Col I) (Figure 6) on osteoblasts has been evaluated in the cytoplasm after 3 days of treatment and showed a similar trend.

As regards SIRT-1, treatment with resveratrol 10 *μ*M (R10), CGF and R10 + CGF upregulated its expression, compared with complete medium (OGM) alone, which represents the control. Also combined treatment with CGF and R10 on osteoblasts treated with zoledronate 5 *μ*M (ZOL5) significantly increased SIRT-1 expression, being statistically different with respect to all treatments, except that with CGF + R10. On the contrary, costimulation with CGF and bisphosphonates significantly reduced SIRT-1 expression with respect to the other treatments. Also, the combined use of CGF and R10 on osteoblasts treated with alendronate 5 *μ*M (AL5) markedly reduced SIRT-1 expression compared with other treatments, except that with CGF + AL5 in which the SIRT-1 expression was significantly increased.

Immunohistochemical analysis for Col I showed that treatment with CGF and CGF + R10 progressively increased Col I expression. In particular, costimulation with CGF and R10 significantly increased Col I compared with OGM, R10, and CGF. On the contrary, the use of R10 did not affect Col I expression that was similar to that of OGM. The addition of CGF to osteoblasts treated with AL5 and ZOL5 significantly reduced Col I levels with respect to osteoblasts treated with CGF and/or CGF + R10. Treatment with CGF + R10 + ZOL5 positively affects Col I expression, being statistically different compared with CGF + AL5. Contrarily, treatment with CGF + R10 + AL5 significantly reduced Col I levels compared with CGF, CGF + R10, and CGF + R10 + ZOL5.

### 3.4. Alizarin Red S Staining

**Figure 7:**
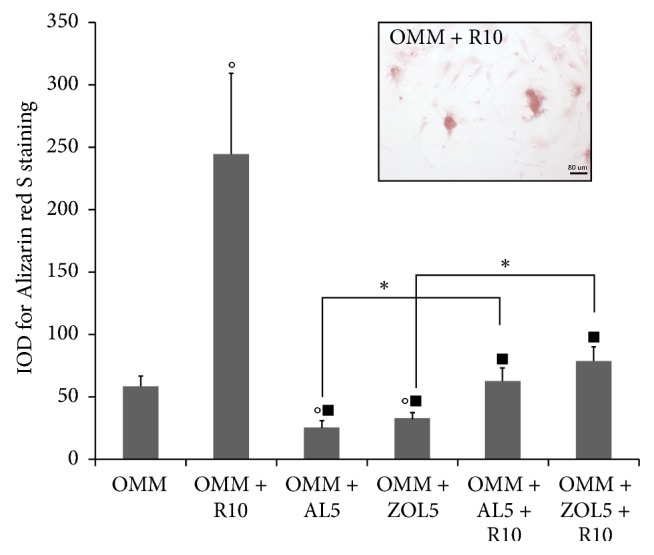
Alizarin Red S staining on osteoblast (HOB) after 21 days of culture as IOD (Integrated Optical Density). A micrograph as an example has been reported. The cells were cultured in mineralization medium (OMM), which represents the control, and in presence of resveratrol 10 *μ*M (R10), alendronate 5 *μ*M (AL5), zoledronate 5 *μ*M (ZOL5), alendronate 5 *μ*M and resveratrol 10 *μ*M (AL5 + R10), and zoledronate 5 *μ*M and resveratrol 10 *μ*M (ZOL5 + R10). Data represent means ± SE, ^*∗*^*p* < 0,05 and in particular °*p* < 0,05 versus OMM; ^*▪*^*p* < 0,05 versus OMM + R10.

Alizarin Red S staining was performed on osteoblasts in mineralization medium (OMM) for 21 days in order to evaluate the degree of calcium deposition. Here, we only report the data obtained for osteoblasts cultured with OMM and also treated with resveratrol 10 *μ*M (R10) (Figure 7) because of the cells treated for 21 days with CGF presenting a red coated surface probably due to haemoglobin deposits which could compromise the correct calcium deposit detection. Results obtained showed that treatment with R10 significantly increased calcium deposition with respect to OMM alone.

## 4. Discussion

The aim of the present study was to evaluate if* in vitro* treatment with the naturally occurring phytoestrogen resveratrol and/or the platelet concentrate CGF could positively influence human osteoblasts proliferation and differentiation. Moreover, we aimed to also investigate whether they could have a protective effect on osteoblasts treated with bisphosphonates. In fact, the first consequence of bisphosphonates chronic administration is a progressive bone degeneration and cellular necrosis at the level of the maxillofacial region, known as bisphosphonate-related osteonecrosis of the jaw or BRONJ [1–3].

The first finding emerging from the present work is that treatment with resveratrol positively affects cell vitality and proliferation. This result is in agreement with previous published data [15, 26, 33] according to which resveratrol promotes bone formation by enhancing the osteoblasts activity and stimulating the osteoblasts proliferation and differentiation. Also, Lin and colleagues [35] showed that daily intake of resveratrol had a beneficial effect on bone turnover in ovariectomized rats, preventing bone loss and suggesting a positive effect of this compound on postmenopausal osteoporosis prevention. Similarly, Feng and collaborators [36] suggest a beneficial effect of resveratrol against osteoporosis. Another important finding emerging from this study is that cotreatment with the platelet concentrate CGF and resveratrol positively influences osteoblasts proliferation and differentiation. In fact, both MTT assay and the evaluation of some osteogenic markers using ELISA and immunohistochemical analysis show a significant osteogenic effect in the presence of CGF and resveratrol 10 *μ*M (R10). Moreover, this treatment seems to have a protective role on osteoblasts treated with bisphosphonates and in particular with the bisphosphonate zoledronate at the final concentration of 5 *μ*M (ZOL5). In fact, MTT assay, ELISA for osteoprotegerin (OPG) after both 3 and 21 days, and immunohistochemistry for sirtuin 1 (SIRT-1) and collagen type I (Col I) show this effect. Regarding bone morphogenetic protein-2 (BMP-2), the treatments with alendronate at the final concentration of 5 *μ*M (AL5) or zoledronate at the final concentration of 5 *μ*M (ZOL5) alone always decreased BMP-2 production at both 3 and 21 days. Moreover, comparing the effects of alendronate and zoledronate on BMP-2 release in different conditions is possible to observe some different results reflecting a different influence of these molecules on osteoblasts. In addition, alendronate and zoledronate influenced differently the osteoblasts cultured in proliferating medium (OGM) with respect to the osteoblasts cultured in differentiating medium (OMM). So, these two factors concurred in the BMP-2 production. At 21 days, R10 was not able to reverse the negative effect of alendronate at the final concentration of 5 *μ*M (AL5) and zoledronate, at the final concentration of 5 *μ*M (ZOL5); only the use of CGF promoted BMP-2 production. Moreover, a possible explanation for the inhibition of BMP-2 production adding resveratrol 10 *μ*M (R10) to zoledronate at the final concentration of 5 *μ*M (ZOL5) plus CGF at 3 days could be related to the balance of the extreme greater enhancement due to CGF addition alone. Moreover, the use of CGF is able to enhance the release of BMP-2 more after 21 days than 3 days with alendronate at the final concentration of 5 *μ*M (AL5), while with zoledronate at the final concentration of 5 *μ*M (ZOL5) the release is enhanced after 3 days with respect to 21 days. Other recent investigation revealed that the suppression of differentiation by zoledronate was associated with decreased expression of BMP-2 and downregulation of the phosphorylation levels in the downstream extracellular signal-regulated kinase 1/2 and p38 pathways [37]. A possible explanation of these results is that the osteogenic effect of resveratrol is probably potentiated by the addition of CGF, containing autologous osteo-inductive growth factors derived from platelets. In particular, it contains and releases some of the principal growth factors involved in bone regeneration, such as bone morphogenetic proteins (BMP), especially BMP-2 and BMP-7, but also TGF-*β*1, PDGF-AB, IGF-1, VEGF, and TNF-*α* [8]. Several studies, both* in vivo* and* in vitro* [38–40], suggest that CGF promotes bone regeneration. Our data show that, in the presence of the bisphosphonate alendronate at the final concentration of 5 *μ*m (AL5), cotreatment with CGF and R10 showed a less pronounced effect except that for BMP-2 release after both 3 and 21 days. In this context, it is important to underline that the exact molecular mechanism of bisphosphonate action is not clear and needed to be further investigated. In fact, several studies have evaluated the effect of alendronate, zoledronate, and other bisphosphonates on osteoblasts proliferation, maturation, and differentiation, obtaining controversial results, depending also on the type of bisphosphonate used as well as on the applied concentration. Koch and colleagues [41] reported an antiproliferative effect of bisphosphonates on osteoblasts, similarly to our MTT results in which bisphosphonates had no significant proliferative effect with respect to OGM and R10. In a similar way, also García-Moreno and collaborators [42] reported that alendronate at high concentrations (>10^-5 ^M) inhibited osteoblasts proliferation, whereas at low concentrations (≤10^-5 ^M) it did not significantly affect osteoblast proliferation compared with controls. Also, Huang and colleagues [43] showed that zoledronate at the micromolar level inhibited osteoblasts proliferation and matrix mineralization. On the contrary, other works [44, 45] suggested that bisphosphonates may stimulate proliferation of osteoblasts and inhibit osteocytes and osteoblasts apoptosis. Also, Im and colleagues [46] showed that alendronate and risedronate promoted proliferation and maturation of both primary osteoblasts and MG-63 osteoblast-like cells.

The second step of the present study was to evaluate the* in vitro* effect of R10, CGF, and cotreatment with both R10 and CGF on the expression of some markers of osteogenesis such as OPG, BMP-2, SIRT-1, and Col I. As regards OPG, bisphosphonates interfere with bone remodeling processes which are controlled by specific mediators such as RANKL, RANK, and OPG. ELISA results showed a different trend in OPG expression after 3 days and 21 days of culture. After 3 days, the highest amount in OPG levels was observed after cotreatment with CGF + R10 + ZOL5 whereas after 21 days in mineralization medium, OPG levels significantly increased in osteoblast treated with CGF, CGF + R10, R10 + ZOL5, and CGF + R10 + ZOL5. These results suggest that R10 and/or CGF also combined with ZOL5 have a positive effect on both osteoblast proliferation and differentiation. Similarly, Koch and colleagues [47] reported that OPG expression was moderately enhanced by zoledronate and ibandronate, after an experimental period of 14 days. Also, the study of Viereck and collaborators [48] reported a significant increase in OPG after 3 days of treatment with zoledronate. Treatment with alendronate instead did not significantly change OPG levels after 3 days of culture but it significantly reduced OPG expression after 21 days in mineralization medium (OMM) in the presence of R10 and/or CGF. These data were in concordance with those reported by Lin and colleagues [49] that did not find a significant influence on OPG expression by alendronate and pamidronate, after 2 days of treatment. Also, in another work [50], it was reported that treatment with alendronate for 24 hours did not have effect on OPG secretion. Given the importance of BMP-2 in osteoblastic differentiation, the present study investigated whether* in vitro* treatment with resveratrol and/or CGF influences the expression of BMP-2. BMP-2 expression showed a different trend after 3 days or 21 days of culture. After 3 days, the highest levels of BMP-2 were detected in cells treated with CGF + ZOL5. Also, cotreatment with CGF + R10 + AL5 and CGF + R10 + ZOL5 increased BMP-2 levels with respect to all treatments, with the exception of CGF + ZOL5. In literature there are different results, which should be clarified. In fact, Ribeiro and colleagues [51] showed an increase of BMP-2 expression with alendronate and zoledronate in cocultured human endothelial and mesenchymal stem cells. Another study [52] demonstrated that* in vitro* zoledronate treatment resulted in a significant upregulation of BMP-2 gene expression, in human osteoblast cells. On the contrary, Huang and colleagues [37] showed that treatment with zoledronate at concentrations ≤ 1 *μ*M significantly decreased BMP-2 expression in a concentration-dependent manner, following 7 days of treatment. After 21 days of treatment in mineralization medium (OMM), high levels of BMP-2 were found in cells treated with CGF alone and combined with R10 and bisphosphonates (AL5 and ZOL5), suggesting that these treatments influenced osteoblast differentiation. As regards CGF, Borsani and collaborators [8] showed that BMP-2 present in this platelet preparation had a slow kinetic release, with a linear increase from 6th day and reaching the maximum accumulation at the end of the experimental period of 8 days. So, probably CGF contributed to osteoblast differentiation. Another study [53] reported that alendronate treatment for 14 days enhanced the differentiation of MG-63 cells, suggesting its role in osteogenic differentiation.

Resveratrol is considered to be an activator of SIRT-1. In fact, Feng and collaborators [36] showed that resveratrol treatment significantly increased the expression of SIRT-1 in a rat model of osteoporosis. Similarly, Zhang and colleagues [54] demonstrated that resveratrol promoted osteoblast proliferation and differentiation by activating SIRT-1. Also, another recent study [23] showed that resveratrol upregulated SIRT-1 levels. Similarly, also results obtained in the present study demonstrated that treatment with resveratrol significantly enhanced SIRT-1 expression, so promoting osteoblast proliferation and differentiation. As showed also treatment with CGF, CGF + R10, and CGF + R10 + ZOL5 significantly increased SIRT-1 levels suggesting that both R10 and CGF may have a protective role on human osteoblasts treated with ZOL5. Similar results were obtained for Col I.

However, the strong limitation of the present study was the deposits observed after the treatment with CGF for 21 days. In fact, this period was too long and the poor availability of donors did not allow replacing CGF more frequently to avoid deposits on cell surface which were not optimal for cell analysis. For this reason, for Alizarin Red staining, we reported data obtained after 21 days of treatment without CGF. Nevertheless, we monitored a recognised marker of osteoblast differentiation, OPG, which could be indicative of* in vitro* bone formation as the alkaline phosphatase (ALP). Indeed, OPG plays a critical role in the regulation of bone turnover [28] and ALP were upregulated in the OPG overexpressing cells [55] demonstrating the link between these two molecules during bone deposition. Moreover, BMP‐2 is one of the most investigated BMP family members and has been identified as the most potent inducer of osteogenesis [29].

## 5. Conclusion

Taken together, the results of this study confirmed that the treatment with resveratrol promotes* in vitro* osteoblast proliferation and differentiation. Moreover, also treatment with CGF and cotreatment with resveratrol and CGF have a protective role on human osteoblasts treated with bisphosphonates and in particular with zoledronate. The use of antioxidants in tissue regeneration has been previously explored, such as the use of platelet concentrates; nevertheless their synergistic effect has been less investigated. Our experiments suggest that the effects of resveratrol on CGF against bisphosphonates-induced side effects are not always synergic and depend on experimental conditions, but resveratrol actually functions as a modulator of CGF effects. These results could be promised in the pathogenesis of BRONJ even if further* in vivo* studies are needed in order to confirm and validate these results.

## Figures and Tables

**Table 1:** Evaluation of working concentration for resveratrol (RSV) after 3 days of treatments using MTT assay.

Cell treatments—3 days	Evaluation performed
Complete medium *(OGM)*—control	MTT for determination of resveratrol (RSV) working concentration
OGM + RSV 1 *µ*M *(R1)*	
OGM + RSV 5 *µ*M *(R5)*	
OGM + RSV 10 *µ*M *(R10)*	
OGM + RSV 25 *µ*M *(R25)*	
OGM + RSV 50 *µ*M *(R50)*	

**Table 2:** Human osteoblast (HOB) treatments in complete medium (OGM) for 3 days.

Cell treatments—3 days	Evaluation performed
Complete medium *(OGM)*—control	MTT ELISA for osteoprotegerin (OPG) and bone morphogenetic protein-2 (BMP-2) immunohistochemistry for sirtuin 1 (SIRT-1) and collagen type I (Col I)
OGM + RSV 10 *µ*M *(R10)*	
OGM + AL 5 *µ*M *(AL5)*	
OGM + ZOL 5 *µ*M *(ZOL5)*	
OGM + AL 5 *µ*M +RSV 10 *µ*M *(AL5 + R10)*	
OGM + ZOL 5 *µ*M +RSV 10 *µ*M *(ZOL5 + R10)*	
OGM + CGF *(CGF) *	
OGM + CGF + RSV 10 *µ*M *(CGF + R10)*	
OGM + CGF + AL 5 *µ*M *(CGF + AL5)*	
OGM + CGF + ZOL 5 *µ*M *(CGF + ZOL5)*	
OGM + CGF + RSV 10 *µ*M + AL 5 *µ*M *(CGF + AL5 + R10)*	
OGM + CGF + RSV 10 *µ*M + ZOL5 *µ*M *(CGF + ZOL5 + R10)*	

**Table 3:** Human osteoblast (HOB) treatments in mineralization medium (OMM) for 21 days.

Cell treatments—21 days	Evaluation performed
Osteoblast mineralization medium *(OMM)*—control	Alizarin Red S staining ELISA for osteoprotegerin (OPG) and bone morphogenetic protein-2 (BMP-2)
OMM + RSV 10 *µ*M *(OMM + R10)*	
OMM + AL 5 *µ*M *(OMM + AL5)*	
OMM + ZOL 5 *µ*M *(OMM + ZOL5)*	
OMM + AL 5 *µ*M +RSV 10 *µ*M *(OMM + AL5 + R10)*	
OMM + ZOL 5 *µ*M +RSV 10 *µ*M *(OMM + ZOL5 + R10)*	
OMM + CGF*(OMM + CGF)*	
OMM + CGF + RSV 10 *µ*M *(OMM + CGF + R10)*	
OMM + CGF + AL 5 *µ*M *(OMM + CGF + AL5)*	
OMM + CGF + ZOL 5 *µ*M *(OMM + CGF + ZOL5)*	
OMM + CGF + RSV 10 *µ*M + AL 5 *µ*M *(OMM + CGF + R10 + AL5)*	
OMM + CGF + RSV 10 *µ*M + ZOL 5 *µ*M *(OMM + CGF + R10 + ZOL5)*	

## References

[1] Graves L. L., Bukata S. V., Aghazadehsanai N., Chang T. I., Garrett N. R., Friedlander A. H. (2016). Patients receiving parenteral bisphosphonates for malignant disease and having developed an atypical femoral fracture are at risk of concomitant osteonecrosis of the jaw: an evidence-based review.

[2] Lundberg A. P., Roady P. J., Somrak A. J., Howes M. E., Fan T. M. (2016). Zoledronate-associated osteonecrosis of the jaw in a dog with appendicular osteosarcoma.

[3] Kharazmi M., Hallberg P., Warfvinge G., Michaëlsson K. (2014). Risk of atypical femoral fractures and osteonecrosis of the jaw associated with alendronate use compared with other oral bisphosphonates.

[4] Rollason V., Laverrière A., Macdonald L. C. I., Walsh T., Tramèr M. R., Vogt-Ferrier N. B. (2016). Interventions for treating bisphosphonate-related osteonecrosis of the jaw (BRONJ).

[5] Del Fabbro M., Gallesio G., Mozzati M. (2015). Autologous platelet concentrates for bisphosphonate-related osteonecrosis of the jaw treatment and prevention. A systematic review of the literature.

[6] Mozzati M., Arata V., Giacomello M. (2015). Failure risk estimates after dental implants placement associated with plasma rich in growth factor-Endoret in osteoporotic women under bisphosphonate therapy.

[7] Bonazza V., Borsani E., Buffoli B., Castrezzati S., Rezzani R., Rodella L. F. (2016). How the different material and shape of the blood collection tube influences the Concentrated Growth Factors production.

[8] Borsani E., Bonazza V., Buffoli B. (2015). Biological characterization and in vitro effects of human concentrated growth factor preparation: An innovative approach to tissue regeneration.

[9] Sohn D.-S., Heo J.-U., Kwak D.-H. (2011). Bone regeneration in the maxillary sinus using an autologous fibrin-rich block with concentrated growth factors alone.

[10] Tadić A., Puskar T., Petronijević B. (2014). Application of fibrin rich blocks with concentrated growth factors in pre-implant augmentation procedures.

[11] Honda H., Tamai N., Naka N., Yoshikawa H., Myoui A. (2013). Bone tissue engineering with bone marrow-derived stromal cells integrated with concentrated growth factor in Rattus norvegicus calvaria defect model.

[12] Yu B., Wang Z. (2014). Effect of concentrated growth factors on beagle periodontal ligament stem cells in vitro.

[13] Ginés C., Cuesta S., Kireev R. (2017). Protective effect of resveratrol against inflammation, oxidative stress and apoptosis in pancreas of aged SAMP8 mice.

[14] Sarubbo F., Esteban S., Miralles A., Moranta D. (2018). Effects of resveratrol and other polyphenols on sirt 1: relevance to brain function during aging.

[15] Dai Z., Li Y., Quarles L. D. (2007). Resveratrol enhances proliferation and osteoblastic differentiation in human mesenchymal stem cells via ER-dependent ERK1/2 activation.

[16] Burns J., Yokota T., Ashihara H., Lean M. E. J., Crozier A. (2002). Plant foods and herbal sources of resveratrol.

[17] Wang Z., Zou J., Huang Y., Cao K., Xu Y., Wu J. M. (2002). Effect of resveratrol on platelet aggregation in vivo and in vitro.

[18] Pagliaro B., Santolamazza C., Simonelli F., Rubattu S. (2015). Phytochemical compounds and protection from cardiovascular diseases: a state of the art.

[19] Zordoky B. N., Robertson I. M., Dyck J. R. (2014). Preclinical and clinical evidence for the role of resveratrol in the treatment of cardiovascular diseases.

[20] Chimento A., Sirianni R., Saturnino C., Caruso A., Sinicropi M. S., Pezzi V. (2016). Resveratrol and its analogs as antitumoral agents for breast cancer treatment.

[21] Rauf A., Imran M., Butt M. S., Nadeem M., Peters D. G., Mubarak M. S. (2016). Resveratrol as an anti-cancer agent: A review.

[22] Loureiro J. A., Andrade S., Duarte A. (2017). Resveratrol and grape extract-loaded solid lipid nanoparticles for the treatment of Alzheimer's disease.

[23] Abed É., Delalandre A., Lajeunesse D. (2017). Beneficial effect of resveratrol on phenotypic features and activity of osteoarthritic osteoblasts.

[24] Ornstrup M. J., Harsløf T., Sørensen L., Stenkjær L., Langdahl B. L., Pedersen S. B. (2016). Resveratrol Increases Osteoblast Differentiation In Vitro Independently of Inflammation.

[25] Guo D. W., Han Y. X., Cong L., Liang D., Tu G. J. (2015). Resveratrol prevents osteoporosis in ovariectomized rats by regulating microRNA-338-3p.

[26] Mizutani K., Ikeda K., Kawai Y., Yamori Y. (2000). Resveratrol attenuates ovariectomy-induced hypertension and bone loss in stroke-prone spontaneously hypertensive rats.

[27] Zhai J.-L., Weng X.-S., Wu Z.-H., Guo S.-G. (2016). Effect of resveratrol on preventing steroid-induced osteonecrosis in a rabbit model.

[28] Simonet W. S., Lacey D. L., Dunstan C. R. (1997). Osteoprotegerin: a novel secreted protein involved in the regulation of bone density.

[29] Kobayashi M., Takiguchi T., Suzuki R., et al (1999). Recombinant human bone morphogenetic protein-2 stimulates osteoblastic differentiation in cells isolated from human periodontal ligament.

[30] Bonazza V., Borsani E., Buffoli B. (2017). In vitro treatment with concentrated growth factors (CGF) and sodium orthosilicate positively affects cell renewal in three different human cell lines.

[31] Lee Y.-M., Shin S.-I., Shin K.-S., Lee Y.-R., Park B.-H., Kim E.-C. (2011). The role of sirtuin 1 in osteoblastic differentiation in human periodontal ligament cells.

[32] Moon J. S., Oh S. H., Jeong Y. W. (2014). Relaxin augments BMP 2-induced osteoblast differentiation and bone formation.

[33] Mizutani K., Ikeda K., Kawai Y., Yamori Y. (1998). Resveratrol stimulates the proliferation and differentiation of osteoblastic MC3T3-E1 cells.

[34] Açil Y., Möller B., Niehoff P. (2012). The cytotoxic effects of three different bisphosphonates in-vitro on human gingival fibroblasts, osteoblasts and osteogenic sarcoma cells.

[35] Lin Q., Huang Y. M., Xiao B. X., Ren G. F. (2005). Effects of resveratrol on bone mineral density in ovarectomized rats.

[36] Feng J., Liu S., Ma S. (2014). Protective effects of resveratrol on Postmenopausal osteoporosis: Regulation of SIRT1-NF-*κ*B signaling pathway.

[37] Huang X., Huang S., Guo F. (2016). Dose-dependent inhibitory effects of zoledronic acid on osteoblast viability and function in vitro.

[38] Chen Y., Cai Z., Zheng D. (2016). Inlay osteotome sinus floor elevation with concentrated growth factor application and simultaneous short implant placement in severely atrophic maxilla.

[39] Qin J., Wang L., Zheng L. (2016). Concentrated growth factor promotes Schwann cell migration partly through the integrin *β*1-mediated activation of the focal adhesion kinase pathway.

[40] Takeda Y., Katsutoshi K., Matsuzaka K., Inoue T. (2015). The Effect of Concentrated Growth Factor on Rat Bone Marrow Cells In Vitro and on Calvarial Bone Healing In Vivo.

[41] Koch F. P., Merkel C., Al-Nawas B. (2011). Zoledronate, ibandronate and clodronate enhance osteoblast differentiation in a dose dependent manner - A quantitative in vitro gene expression analysis of Dlx5, Runx2, OCN, MSX1 and MSX2.

[42] García-Moreno C., Serrano S., Nacher M. (1998). Effect of alendronate on cultured normal human osteoblasts.

[43] Huang K.-C., Cheng C.-C., Chuang P.-Y., Yang T.-Y. (2015). The effects of zoledronate on the survival and function of human osteoblast-like cells.

[44] Maruotti N., Corrado A., Neve A., Cantatore F. P. (2012). Bisphosphonates: Effects on osteoblast.

[45] Corrado A., Neve A., Maruotti N., Gaudio A., Marucci A., Cantatore F. P. (2010). Dose-dependent metabolic effect of zoledronate on primary human osteoblastic cell cultures.

[46] Im G. I., Qureshi S. A., Kenney J., Rubash H. E., Shanbhag A. S. (2004). Osteoblast proliferation and maturation by bisphosphonates.

[47] Koch F. P., Merkel C., Ziebart T., Smeets R., Walter C., Al-Nawas B. (2012). Influence of bisphosphonates on the osteoblast RANKL and OPG gene expression in vitro.

[48] Viereck V., Emons G., Lauck V. (2002). Bisphosphonates pamidronate and zoledronic acid stimulate osteoprotegerin production by primary human osteoblasts.

[49] Lin J. M., Callon K. E., Lin C. Q. (2007). Alteration of bone cell function by RANKL and OPG in different in vitro models.

[50] Giner M., Rios M. J., Montoya M. J., Vázquez M. A., Miranda C., Pérez-Cano R. (2011). Alendronate and raloxifene affect the osteoprotegerin/RANKL system in human osteoblast primary cultures from patients with osteoporosis and osteoarthritis.

[51] Ribeiro V., Garcia M., Oliveira R., Gomes P. S., Colaço B., Fernandes M. H. (2014). Bisphosphonates induce the osteogenic gene expression in co-cultured human endothelial and mesenchymal stem cells.

[52] Pan B., To L. B., Farrugia A. N. (2004). The nitrogen-containing bisphosphonate, zoledronic acid, increases mineralisation of human bone-derived cells in vitro.

[53] Xiong Y., Yang H. J., Feng J., Shi Z. L., Wu L.-D. (2009). Effects of alendronate on the proliferation and osteogenic differentiation of MG-63 cells.

[54] Zhang P., Li Y., Du Y., Li G., Wang L., Zhou F. (2016). Resveratrol Ameliorated Vascular Calcification by Regulating Sirt-1 and Nrf2.

[55] Yu H., de Vos P., Ren Y. (2011). Overexpression of osteoprotegerin promotes preosteoblast differentiation to mature osteoblasts.

